# Prognostic Value of Metastatic No.8p LNs in Patients with Gastric Cancer

**DOI:** 10.1155/2015/937682

**Published:** 2015-11-16

**Authors:** Dong-Jiao Guo, Kun Yang, Wei-Han Zhang, Xiao-Long Chen, Xin-Zu Chen, Bo Zhang, Zong-Guang Zhou, Jian-Kun Hu

**Affiliations:** Department of Gastrointestinal Surgery, West China Hospital, Sichuan University, Chengdu, Sichuan 610041, China

## Abstract

*Background*. To evaluate prognostic value of metastatic No.8p LNs in patients with gastric cancer. *Methods*. From August 2002 to December 2011, a total of 284 gastric cancer patients who underwent gastrectomy with No.8p LNs dissection were analyzed retrospectively in this study. Patients were divided into two groups according to the status of No.8p LNs. Clinicopathological features were collected to conduct the correlation analysis. Follow-up was carried out up to December 31st, 2014. Overall survival was analyzed. *Results*. Out of 284 patients, metastatic No.8p LNs were found in 24 (8.5%) patients. Compared with other 260 cases, these patients suffered morphologically larger tumor (*P* = 0.003), node stage (*P* = 0.000), and metastatic stage (*P* = 0.000). The 3-year overall survival rate was 26% in No.8p-positive group and 53% in No.8p-negative group. No significant difference of cumulative survival rates existed between the No.8p-positive group and No.8p-negative stage IV group (26% versus 28%, *P* = 0.923). Patients with other distant metastasis or not in No.8p+ group had similar cumulative survival rates (24% versus 28%, *P* = 0.914). *Conclusions*. Positive No.8p LNs were a poor but not an independent prognostic factor for patients with GC and should be recognized as distant metastasis.

## 1. Introduction

Gastric cancer is the second most frequent diagnosed cancer and one of the most common death-leading cancers in the world [1–4]. Gastrectomy plus appropriate lymphadenectomy is the primary treatment for resectable gastric cancer. However, there remains controversy about the degree of lymph node dissection. Two European large-scale randomized controlled clinical trials failed to prove that D2 lymphadenectomy outweighed D1 lymphadenectomy [5–8], partially due to the increased postoperative morbidity, mortality, and reoperation rate without increasing survival rate. Then, further studies revealed that D2 lymphadenectomy was also associated with lower local-regional recurrence, gastric-cancer-related death, and a better survival benefit [9–11]. However, Jiang et al. conducted a meta-analysis of 12 randomized controlled trials that showed no better overall survival benefit from D2 lymphadenectomy than that of D1 lymphadenectomy [12]. Therefore, the current consensus in West countries is gastrectomy plus D1 or modified D2 lymphadenectomy for gastric cancer [2, 13]. While clinical experience from observational or randomized controlled trails in Asia demonstrated D2 lymphadenectomy could lead to better outcomes than D1 lymphadenectomy. Thus D2 lymphadenectomy is recommended as the standard procedure for resectable gastric cancer according to the treatment guideline of Japanese Gastric Cancer Association (JGCA) [14]. As for D2 lymphadenectomy plus the extraregional lymph nodes (e.g., No.13 LNs or No.16 LNs), the results of previous studies were not cogent enough because of their own limitations [15, 16]. No.8p LNs were defined as posterior lymph nodes along the common hepatic artery and also classified as the extraregional lymph nodes [17]. It had long been theorized that prognosis of patients with evident metastatic No.8p LNs was poor, but little data was available. In this retrospective study, we aimed to analyze the prognostic value of No.8p LNs in patients with gastric cancer.

## 2. Method

### 2.1. Patients

From August 2002 to December 2011, clinicopathological and survival data of 284 GC patients who underwent total or subtotal gastrectomy with D1+ or D2+ lymphadenectomy plus No.8p LNs dissection in Department of Gastrointestinal Surgery, West China Hospital, Sichuan University, were retrospectively analyzed. Patients were included in this study based on the following principles: (1) preoperatively histological confirmation of gastric adenocarcinoma, (2) gastrectomy with lymphadenectomy plus No.8p LNs dissection, and (3) no remnant gastric cancer patients. Patients were divided into the No.8p-positive group (No.8p+ group) and the No.8p-negative group (No.8p− group) according to No.8p LNs status reported in postoperative pathology.

### 2.2. Extent of Lymphadenectomy

D1+ lymphadenectomy was regarded as gastrectomy with extended lymphadenectomy exceeding D1 but not reaching D2. While D2+ lymphadenectomy was defined as gastrectomy with lymphadenectomy beyond D2, such as lymph nodes around the area of posterior surface of the pancreatic head (No.13), superior mesenteric vein (No.14v), or the para-aortic (No.16), and so forth. The principles above were applied according to Japanese Gastric Cancer Treatment Guidelines [14]. In this study, all cases underwent D1+ or D2+ lymphadenectomy plus No.8p LNs dissection.

After the dissection of suprapyloric lymph nodes, No.8a LNs were gripped in the root of arteria gastroduodenalis. The contour of the common hepatic artery was confirmed before it was barred from its initial to the proper hepatic artery. Then No.8p LNs could be dissected in vivo by the operating surgeon. The surgery related data was recorded in the advanced database of the department.

### 2.3. Clinicopathological Data

The clinicopathological features contained age, gender, tumor location (longitudinal and cross-section location), histological and macroscopic type, number of harvested and metastatic lymph nodes, and tumor stage. The histological types were categorized into differentiated type and undifferentiated type. The former consisted of well, moderate, and poor differentiated adenocarcinoma, while the latter was made up of signet-ring cell carcinoma, mucinous adenocarcinoma, papillary adenocarcinoma, and undifferentiated adenocarcinoma. Tumor staging was conducted according to the tumor-node-metastasis system of Japanese Gastric Cancer Association [17].

### 2.4. Follow-Up

Patients underwent regular follow-up through outpatient visit, mails, or telephones. The last follow-up was updated to December 31st, 2014. The follow-up time ranges from 36 months to 116 months. 21 cases were lost to follow-up and the lost rate was 14.4%. Overall 3-year survival (OS) rate was evaluated in this study.

### 2.5. Statistical Analysis

Continuous variables were presented as mean ± standard deviation and analyzed using with the Mann-Whitney *U* test. Categorical data was analyzed by the means of the Chi-square test or Wilcoxon test as appropriate. The risk factors of No.8p LNs metastasis were analyzed by Rank-Sum test and Chi-square test for univariate analysis and logistic regression for multivariate analysis. OS curves of patients between subgroups were calculated by Kaplan-Meier method from the day of operation to the final follow-up or death, and differences between the survival curves were assessed by log-rank test. Cox proportional hazards model was used to identify prognostic factors in univariate and multivariate analysis. The two side's *P* value <0.05 was considered as statistic significant. Statistical analysis was conducted by the Statistical Package for Social Science version 19.0 (SPSS, Chicago, IL, USA).

## 3. Results

### 3.1. Correlation Analysis between Clinicopathological Features and Metastasis of No.8p LNs

24 cases of 284 patients (8.5%) showed positive metastasis of No.8p LNs. Clinicopathological features of patients were analyzed between the two groups (Table 1). Significant differences were found in tumor diameter (*P* = 0.003), mean number of metastatic lymph nodes (*P* = 0.000), types of gastrectomy (*P* = 0.042), and curative degrees (*P* = 0.000) owing to M1 disease, but no statistic differences were found in age (*P* = 0.685), gender (*P* = 0.840), tumor location (*P* > 0.05), mean number of harvested lymph nodes (*P* = 0.333), macroscopic types (*P* = 0.574), differentiation grade (*P* = 0.292), and lymphadenectomy (*P* = 0.085). Moreover, the lymph node metastatic ratio was 57.0% in the No.8p+ group and 19.3% in the No.8p− group (*P* < 0.001). Patients suffered more advanced T stages (*P* = 0.024), N stages (*P* = 0.000), and M stages (*P* = 0.000) in No.8p+ group than these of No.8p− group (Table 2).

Logistic regression verified that metastasis of No.8p LNs was closely related to positive No.8a LNs (hazard ratio [HR], 4.437; *P* = 0.040) compared with regional lymph nodes, other extraregional lymph nodes (e.g., No.13, No.15, and No.16), tumor location.

### 3.2. Morbidity and Mortality

No patient died within postoperative 30 days. No difference existed among operating time, intraoperative blood loss, and postoperative hospital stay between the two groups (Table 3). In the No.8p− group, the most common complications were gastroparesis (1.2%), followed by paralytic intestinal obstruction (0.8%), fistula (0.4%), abdominal hemorrhage (0.4%), and intra-abdominal infection (0.4%). Only one case of anastomosis fistula (4.1%) was found in No.8p+ group.

### 3.3. Survival Outcomes and Variate Analysis

**Figure 1:**
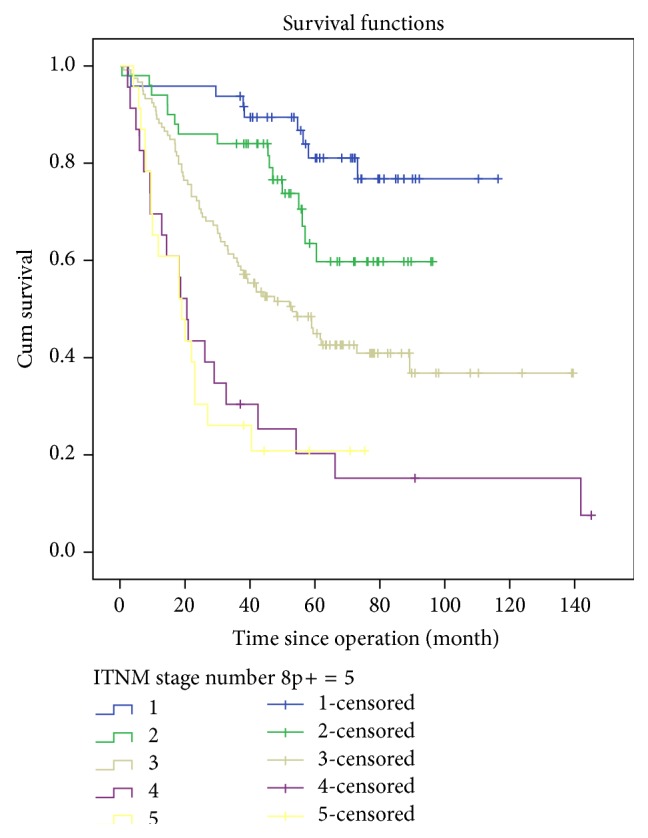
Cumulative survival rates categorized by tumor stage and No.8p status. No.8p-negative in stage IV versus No.8p-positive, *P* = 0.923. No.8p-negative in stage I/II/III versus No.8p-positive, *P* < 0.050. log rank test. pTNM stage is based on the Japanese classification of gastric carcinoma: 3rd English edition.

**Figure 2:**
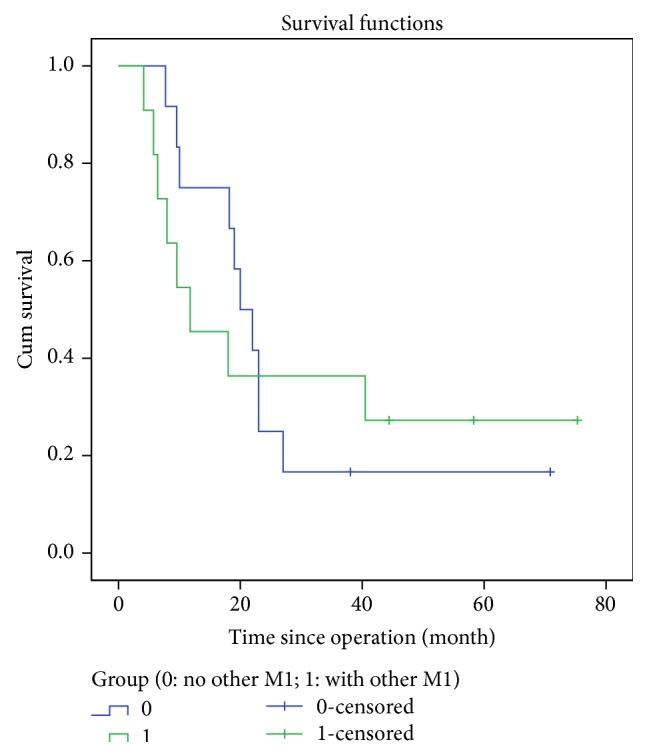
Cumulative survival rates categorized by distant metastasis in No.8p-positive group, *P* = 0.914, No.8p-positive group without other M1 versus No.8p-positive group with other M1. log rank test. pTNM stage is based on the Japanese classification of gastric carcinoma: 3rd English edition.

Overall 3-year survival rate was 26.0% in No.8p+ group and 53.0% in No.8p− group (*P* = 0.005). We mainly explored the comparison of survival outcomes in No.8p− group at stage III/IV, because patients at stage I/II in No.8p− group did not reach their median survival time until the latest follow-up (Table 4). Significant difference of 3-year overall survival rates of the two groups existed in the items of gender, age gastrectomy, pathological degree, and curative degree (*P* < 0.050). Univariate analysis revealed that R1/R2 (*P* = 0.000), subtotal gastrectomy (*P* = 0.007), advanced T stage (*P* < 0.050), distant metastasis (*P* = 0.000), and positive No.8p LNs (*P* = 0.000) brought about higher risks of worse overall survival in GC patients, while multivariate analysis also illustrated R1/R2, T4 stage and N3b stage could run higher risks of worse overall survival in GC patients (*P* < 0.050) (Table 5). Moreover, the cumulative survival rate of No.8p− group in stage IV showed no statistical difference from that of No.8p+ group (*P* = 0.923). The cumulative survival rates of No.8p− group in stage I/II/III presented statistical difference from that of No.8p+ group (*P* < 0.050) (Figure 1). Patients in the No.8p+ group showed no statistical difference of cumulative survival rates, whether they had other extraregional lymph nodes or not (*P* = 0.914) (Figure 2).

## 4. Discussion

Extent of lymph node dissection has been a decade-old argument, since D2 lymphadenectomy was recommended to be performed by experienced surgeons in West and the standard procedure in Japan [5–14]. Some of the extraregional lymph nodes had been reported [15, 16], but the prognostic value of No.8p LNs dissection is still unclear. Our study showed that positive No.8p LNs should be defined as distant metastasis rather than regional lymph node metastasis and positive No.8p LNs was a poor prognostic factor for GC patients.

Maruyama et al. reported that the incidence of metastasis of No.8 LNs was about 16% [18], and Sasako et al. reported that the therapeutic index of No.8 LNs was 5.9 by multiplying the metastatic frequency of No.8 LNs with the 5-year survival rate of patients with positive No.8 LNs [19]. The two studies focus mainly on No.8a LNs because of fewer metastasis of No.8p LNs. Based on this kind of therapeutic index and lymphatic flow at different tumor sites, No.8p LNs were recognized as the extraregional lymph nodes [17]. In this study, we confirmed that metastasis rate of No.8p LNs was 8.5%, which was consistent with previous reports [18, 19]. But we failed to calculate the therapeutic index of No.8p LNs as Sasako et al. did, mainly because of the short follow-up time and relative small sample size. Hence, more high-quality and large-sample trials and long-term follow-up are necessary to assess the therapeutic index of No.8p LNs. Another characteristic, we need to notice, was the metastasis of No.8p LNs that was closely related to that of No.8a LNs compared with the other lymph nodes, tumor location. This might be in accordance with their anatomical relationship and lymphatic flow.

Patients in the No.8p+ group suffered more advanced pathological stage. Moreover, patients in the No.8p− group enjoyed better survival in the I/II/III stage than those in the No.8p+ group (*P* < 0.050), but patients in the No.8p− group showed no survival difference in the IV stages compared with patients in the No.8p+ group (*P* = 0.923). Even after R0 resection, the 3-year survival rate in No.8p+ group was much lower than that in No.8p− group (*P* = 0.003). Moreover, lymph node metastatic ratio had been advocated to be a more appropriate method for N stage and predicted survival partly in recent years [20, 21]. From our study, patients in the No.8p+ group had a higher lymph node metastatic ratio than that in the No.8p− negative group (*P* < 0.001). At pN3 stage, the lymph node metastatic ratio in the No.8p+ group was 0.446 at pN3a stage and 0.740 at pN3b stage, respectively. We also demonstrated that the 3-year survival rate in the No.8p+ group was 50.0% at pN3a stage and 8.0% at pN3b stage, respectively. Moreover, univariate analysis revealed positive No.8p LNs (*P* = 0.000) brought about higher risks of worse overall survival, while multivariate analysis illustrated that positive No.8p LNs was not an independent prognostic factor in GC patients. All the factors above demonstrated that No.8p LNs were extraregional lymph nodes rather than regional lymph nodes and that metastasis of No.8p LNs should be recommended as distant metastasis, which was in accordance with the Japanese Classification of Gastric Carcinoma [17].

## 5. Conclusion

It was acknowledged that the number of cases was small in No.8p+ group. Higher rate of other M1 diseases made R0 rate less in No.8p+ group. However, survival outcomes between cases in No.8p+ group and IV stage cases in No.8p− group indicated that positive No.8p LNs was a poor but not an independent prognostic factor for patients with gastric cancer and should be recognized as the distant metastasis rather than regional lymph node metastasis.

## Figures and Tables

**Table 1:** Details of clinicopathological characteristics and univariate correlation analysis of No.8p LNs.

	Characteristics	No.8p LNs (positive)	No.8p LNs (negative)	*P* value
		*n* = 24 (%)	*n* = 260 (%)	
Age	Years	54.8 ± 15.0 (27–85)	55.8 ± 11.6 (26–80)	0.685^∗^
	>60	8 (33.3)	105 (40.4)	0.499
	≤60	16 (66.7)	155 (50.6)	
Gender	Male	16 (66.7)	168 (64.6)	0.840
	Female	8 (33.3)	92 (35.4)	
Longitudinal location	U	5 (20.8)	61 (23.5)	0.263
	M	7 (29.2)	60 (33.1)	
	L	11 (45.8)	134 (51.5)	
	Combined	1 (4.2)	5 (1.9)	
Cross-sectional location	Lesser	11 (45.8)	130 (50.0)	0.914
	Greater	4 (16.7)	30 (11.5)	
	Anterior	1 (4.2)	13 (5.0)	
	Posterior	1 (4.2)	19 (7.3)	
	Multiwalls	7 (29.2)	68 (26.2)	
Differentiation grade^†^	Differentiated	5 (20.8)	81 (31.2)	0.292
	Undifferentiated	19 (79.2)	179 (68.3)	
Diameter (cm)	Mean ± SD	7.06 ± 4.31	5.06 ± 3.00	0.003^∗^
	EGC	0 (0)	8 (3.1)	0.574
Macroscopic type	Borrmann-I	1 (4.2)	10 (3.8)	
	Borrmann-II	11 (45.8)	145 (55.8)	
	Borrmann-III	8 (33.3)	75 (28.8)	
	Borrmann-IV	4 (26.7)	22 (8.5)	
Metastatic LNs	Number	18.5 ± 11.8	5.2 ± 6.4	0.000^∗^
Harvested LNs	Number	31.0 ± 11.9	33.5 ± 12.1	0.333^∗^
LN metastatic ratio	Percent	57.0	19.3	<0.001
Gastrectomy	Total	13	84	0.042
	Subtotal	11	176	
Curative degree	R0	16 (66.7)	237 (91.2)	0.000
	R1/R2	8 (33.3)	23 (8.8)	
Lymph node resection	D1+	10 (41.7)	66 (25.4)	0.085
	D2+	14 (58.3)	194 (74.6)	

^∗^Student's *t*-test. ^†^Histologic differentiation grade is based on the Japanese classification of gastric carcinoma: 3rd English edition

EGC: early gastric cancer.

**Table 2:** Details of pathological stage and univariate correlation analysis of No.8p LNs.

	Characteristics	No.8p+ group	No.8p− group	*P* value
		*n* = 24 (%)	*n* = 260 (%)	
T stage	T1	1 (4.2)	45 (17.3)	0.024
	T2	0 (0)	36 (13.8)	
	T3	1 (4.2)	21 (8.1)	
	T4	22 (91.6)	158 (60.8)	

N stage	N0	0 (0.0)	82 (31.5)	0.000
	N1	0 (0.0)	47 (18.1)	
	N2	2 (8.3)	52 (20.0)	
	N3a	9 (37.5)	56 (21.5)	
	N3b	13 (54.2)	23 (8.8)	

M stage	M0	0 (0)	236 (90.8)	0.000
	M1^∗^	24 (100.0)	24 (9.2)	

pTNM stage	IA	0 (0)	36 (13.8)	0.000
	IB	0 (0)	17 (6.5)	
	IIA	0 (0)	23 (8.8)	
	IIB	0 (0)	30 (11.5)	
	IIIA	0 (0)	34 (13.1)	
	IIIB	0 (0)	46 (17.7)	
	IIIC	0 (0)	48 (18.5)	
	IV	24 (100.0)	26 (10.0)	

pTNM stage is based on the Japanese classification of gastric carcinoma: 3rd English edition.

^∗^M1 include positive extraregional lymph nodes (*n* = 13), peritoneal metastasis (*n* = 9), hepatic metastases (*n* = 1), and Krukenberg tumor (*n* = 1).

**Table 3:** Comparison of morbidity and mortality between No.8p+ and No.8p− groups.

	Characteristics	No.8p+ group	No.8p− group	*P* value
		*n* = 24 (%)	*n* = 260 (%)	
Operating time	min	249 ± 41	260.1 ± 48.9	0.457
Intraoperative blood loss	mL	161 ± 104	168 ± 97	0.295
Postoperative hospital stay	Day	11 ± 5	11 ± 4	0.961
Overall complications	Anastomosis fistula	0	1	0.717
	Lymphatic chylous fistula	1	0	
	Paralytic intestinal obstruction	0	1	
	Abdominal hemorrhage	0	1	
	Intra-abdominal infection	0	3	
	Gastroparesis	1	14	
	Others^∗^	0	2	

^∗^Others include pulmonary infection (*n* = 1), delirium (*n* = 1).

**Table 4:** The comparison of GC patient survival outcomes in No.8p+ group and in No.8p− group at stage III/IV.

	No.8p+ group	No.8p− group	*P* value
	3-year OS (%)	3-year OS (%)	
Total	26.0	53.0	0.005
Gender			0.040
Male	27.0	51.0	
Female	25.0	56.0	
Age (years)			0.011
>60	14	40	
≤60	31	61	
Gastrectomy			
Total Gastrectomy	25	61	0.006
Subtotal gastrectomy	27	43	
Pathological degree			
Differentiated	33	55	0.041
Undifferentiated	10	47	
Curative degree			0.003
R0	19	55	
R1/R2	0	40	
Lymphadenectomy			
D1+	22	51	0.115
D2+	29	57	

pTNM stage in the No.8p− group is based on the Japanese classification of gastric carcinoma: 3rd English edition.

**Table 5:** Univariate and multivariate analysis of prognostic factors on overall survival in patients gastric cancer based on Cox proportional hazards model.

Variables	Univariate	*P* value	Multivariate	*P* value
	HR (95% CI)		HR (95% CI)	
Age (years)				
>60	1			
≤60	0.742 (0.524, 1.050)	0.092		
Gastrectomy				
Subtotal gastrectomy	1		1	
Total gastrectomy	1.778 (1.251, 2.252)	0.001	1.166 (0795, 1.710)	0.443
Tumor location				
Upper	1			
Middle	0.652 (0.384, 1.107)	0.113		
Lower	0.536 (0.363, 0.790)	0.002		
Total	2.154 (0.772, 6.010)	0.083		
Pathological degree				
Differentiated	1			
Undifferentiated	0.949 (0.653, 1.378)	0.782		
Curative degree				
R0	1		1	
R1/R2	3.926 (2.095, 7.356)	0.000	2.452 (1.267, 4.746)	0.008
Lymphadenectomy				
D1+	1			
D2+	1.215 (0.810, 1.823)	0.347		
T stage				
T1	1		1	
T2	3.257 (1.147, 9.246)	0.027	2.514 (0.856, 7.387)	0.094
T3	3.697 (1.170, 11.687)	0.026	2.724 (0.833, 8.911)	0.097
T4	7.791 (3.173, 19.128)	0.000	4.556 (1.725, 12.035)	0.002
N stage				
N0	1		1	
N1	1.513 (0.798, 2.870)	0.205	1.024 (0.527, 1.990)	0.943
N2	1.935 (1.082, 3.461)	0.026	1.124 (0.605, 2.088)	0.711
N3a	3.429 (2.110, 7.103)	0.000	1.771 (0.986, 3.182)	0.056
N3b	7.245 (4.075, 12.880)	0.000	3.644 (1.952, 6.801)	0.000
M stage				
M0	1		1	
M1	3.223 (2.137, 4.861)	0.000	1.551 (0.877, 2.744)	0.132
No.8p LNs status				
Negative	1		1	
Positive	3.101 (1.873, 5.136)	0.000	0.892 (0.469, 1.696)	0.728

Backward variable selection with selection criteria of 0.2 was conducted with all clinicopathologic variables. pTNM stage is based on the Japanese classification of gastric carcinoma: 3rd English edition.
